# Different gene expression profiles in normo- and dyslipidemic men after fish oil supplementation: results from a randomized controlled trial

**DOI:** 10.1186/1476-511X-11-105

**Published:** 2012-08-29

**Authors:** Simone Schmidt, Frank Stahl, Kai-Oliver Mutz, Thomas Scheper, Andreas Hahn, Jan P Schuchardt

**Affiliations:** 1 Institute of Food Science and Human Nutrition, Leibniz University of Hannover, Am Kleinen Felde 30, 30167, Hannover, Germany; 2 Institute of Technical Chemistry, Leibniz University of Hannover, Callinstr. 5, 30167, Hannover, Germany

**Keywords:** Lipid metabolism, Dyslipidemia, Hypertriglyceridemia, Pathway analysis, Cardiovascular disease, Gene regulation, Genome microarrays, Omega-3 fatty acids, Omega-3 index

## Abstract

**Background:**

Epidemiological studies have suggested the benefits of omega-3 polyunsaturated fatty acids (n-3 PUFAs) on cardiovascular health, but only limited data are available describing n-3 PUFA regulated pathways in humans. The aim of this study was to investigate the effects of n-3 PUFA administration on whole genome expression profiles in the blood of normo- and dyslipidemic subjects.

**Methods:**

Differentially expressed genes were detected after four hours, one week and twelve weeks of supplementation with either fish oil (FO) or corn oil in normo- and dyslipidemic men using whole genome microarrays.

**Results:**

Independent of the oil, a significantly higher number of genes was regulated in dyslipidemic subjects compared to normolipidemic subjects. Pathway analyses discovered metabolisms dominantly affected by FO after twelve weeks of supplementation, including the lipid metabolism, immune system and cardiovascular diseases. Several pro-inflammatory genes, in particular, were down-regulated in dyslipidemic subjects, indicating the immune-modulatory and anti-inflammatory capability of FO and its bioactive FAs, eicosapentaenoic acid and docosahexaenoic acid.

**Conclusions:**

This is the first study showing significant differences in gene expression profiles between normo- and dyslipidemic men after FO supplementation. Further studies need to clarify the exact role of n-3 PUFAs in pathways and metabolisms which were identified as being regulated after FO supplementation in this study.

**Trial registration:**

ClinicalTrials.gov (ID: NCT01089231)

## Background

Numerous epidemiologic and intervention studies have shown the beneficial effects of fish oil (FO) and its principal omega-3 poly unsaturated fatty acids (n-3 PUFAs), eicosapentaenoic acid (EPA, 20:5n-3) and docosahexaenoic acid (DHA, 22:6n-3), in the prevention of atherosclerosis and cardiovascular diseases (CVD) [1-3]. EPA and DHA are known to affect the lipid profile by reducing the elevated triacylglycerol (TG) level and increasing the high density lipoprotein (HDL) level [4,5]. Due to the effects on lipid levels, subjects with dyslipidemia, especially hypertriglyceridemia, benefit from FO supplementation to reduce the cardiovascular risk [6-8].

Beyond the positive effects on lipid levels, other cardio-protective effects of EPA and DHA are known involving anti-inflammation, modulation of cardiac ion channels, influence on membrane microdomains and downstream cell signaling pathways, anti-thrombotic and anti-arrhythmic effects, induction of hemodynamic changes, and improvement of endothelial function [9,10]. The underlying molecular mechanisms by which EPA and DHA exert these beneficial effects on cardiovascular health are not completely understood. It is believed that many effects of n-3 PUFAs are mediated by interferences with signaling transduction pathways [11].

N-3 PUFAs are known to affect a myriad of molecular pathways, including the regulation of gene expression [12]. This regulation can be driven by n-3 PUFAs directly, or by their secondary metabolites, for example, eicosanoids. It is known that n-3 PUFAs induce changes in the expression of several genes related to lipid and carbohydrate metabolism, cell differentiation and growth, cytokine, adhesion, and eicosanoid production, as well as oxidative and immune system processes [13-15]. The inhibition of inflammatory signaling, observed in *in vitro* and animal studies, is viewed as one of the major mechanisms on how n-3 PUFAs may improve cardiovascular health [11]. However, the anti-inflammatory effect of n-3 PUFAs has not been completely confirmed in human subjects [10].

Only a few human studies have investigated genome-wide expression changes after FO supplementation to identify specific metabolic pathways. Bouwens and co-workers analyzed the effects of FO supplementation on whole genome expression changes and performed several pathway analyses which mainly showed a down-regulation of genes involved in inflammatory and stress-related pathways [16]. Another study monitored the expression changes of 588 genes after FO supplementation and discovered a regulative effect in several lymphocyte functions such as signaling, cell cycle, cytokine production, and apoptotic and stress response [17].

To our knowledge, gene expression profiles in response to FO supplementation have not been investigated in dyslipidemic subjects so far. Connections between dyslipidemia, especially hypertriglyceridemia and HDL-hypercholesterolemia, and chronic inflammation have already been uncovered [18]. The discovery of specific FO metabolic pathways which are influenced by FO in dyslipidemic states may provide insights into the anti-inflammatory and lipid-lowering effects of n-3 PUFAs. In addition, it is not known if normo- and dyslipidemic subjects display different gene expression profiles in response to FO supplementation. Accordingly, we conducted a nutrition study to identify the differences in gene expression profiles after FO supplementation between normo- and dyslipidemic subjects.

## Methods

A randomized, double-blind, controlled, parallel intervention study of three months duration was undertaken. This investigator-initiated study was designed and conducted according to the principles of the Good Clinical Practice Guidelines laid down in the Declaration of Helsinki. The study was registered at ClinicalTrials.gov (ID: NCT01089231).

### Subjects

**Figure 1:**
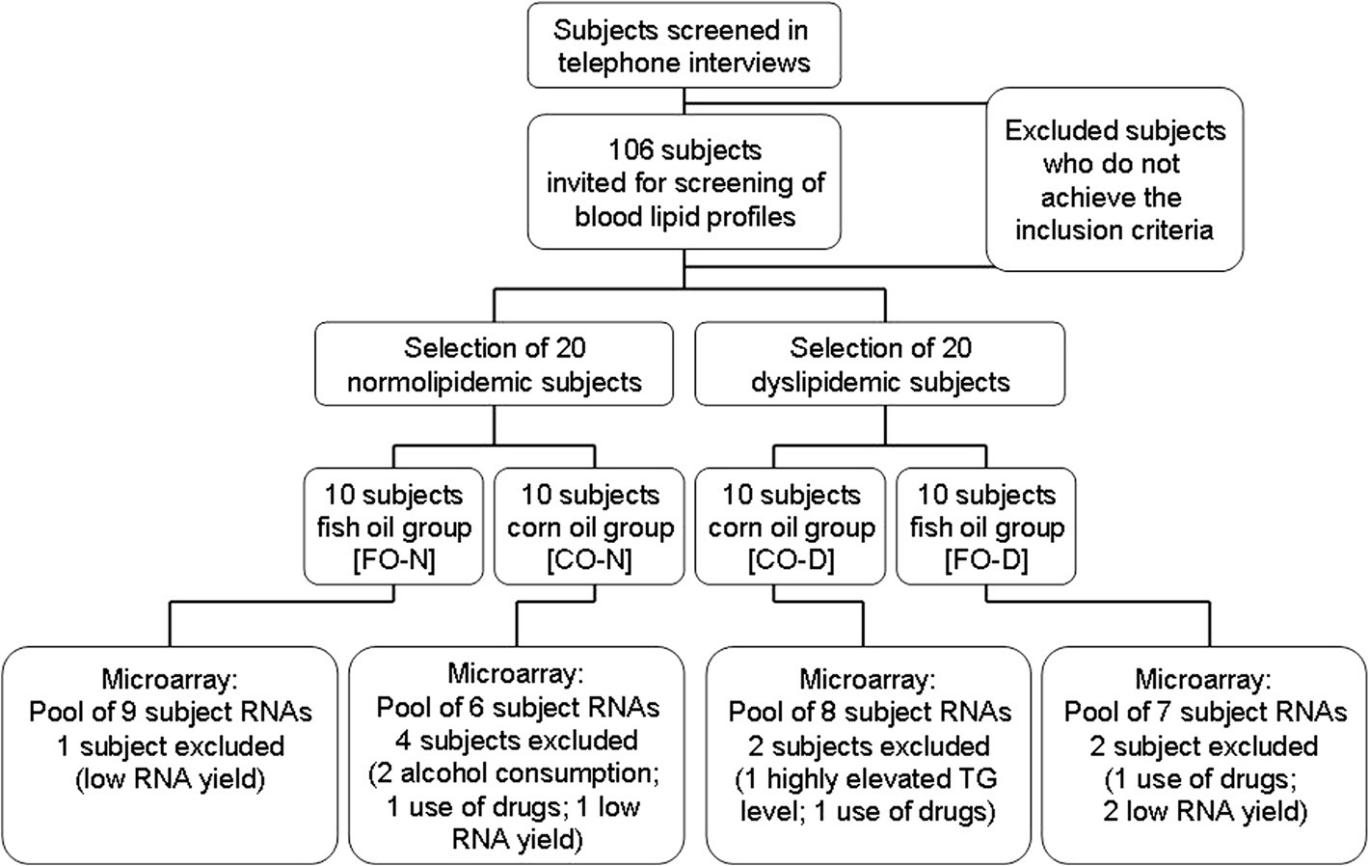
Flow chart of subject recruitment and sample selection for microarray analysis.

The recruitment of subjects was performed by several advertisements and study placards in Hannover. One hundred and six subjects were pre-selected via telephone interviews according to the following exclusion criteria: Female; body-mass-index > 35; smoker; intake of any corticosteroid, lipid-lowering or anti-inflammatory drugs; diagnosed chronic - cardiovascular - or liver diseases; gastrointestinal disorders; blood coagulation disorders and intake of coagulation-inhibiting drugs (e.g. Marcumar); renal failure; periodic intake of laxatives; ingestion of supplements enriched with n-3 PUFAs, phytosterols, polyglucosamines, other lipid-binding ingredients, or daily eating of fatty fish; allergy to fish or FO; or participation in another clinical study < 30 days before the start of the study or at the same time. The pre-selected subjects were invited for a screening examination to collect fasting blood and determine serum lipid levels. Among these subjects, 20 normolipidemic men (total cholesterol (TC) < 200 mg/dl; low density lipoprotein (LDL) < 130 mg/dl; TG < 150 mg/dl) and 20 dyslipidemic men (TC > 200 mg/dl; LDL > 130 mg/dl; TG > 150 mg/ml), aged between 29 and 51 years, were enrolled in the study population (Figure 1). All subjects included gave their written informed consent to take part in the study, which was approved by the Freiburger ethics committee.

### Study design

The 20 normolipidemic and the 20 dyslipidemic subjects were subdivided into two groups. Thus, a total of four groups with ten men per group passed through the study. The formation of groups was performed by stratified allocation according to each subject’s age to realize a comparable mean age between the groups. The four study groups were randomly assigned to different study products by an uninvolved collaborator. Subjects ingested either six FO or six corn oil (CO) capsules per day for a period of twelve weeks. The daily n-3 PUFA intake via FO capsules was 2.7 g (1.14 g DHA and 1.56 g EPA). The predominant FA of the CO capsules was the omega-6 (n-6) PUFA linoleic acid (LA, 18:2n-6). Thus, the daily LA intake via CO capsules was 3.05 g LA. The subjects were instructed to ingest the capsules together with food, three in the morning and three in the evening, and to maintain their usual exercise and dietary habits throughout the intervention time. As an exception, on the first intervention day, all six capsules were ingested at the same time in the morning after a standardized breakfast. Fasting blood samples were collected by venepuncture during each visit. Additionally, participants completed a questionnaire to obtain information about changes in medication, diet (e.g. changes in weekly fish intake, preferred fish dishes or species, respectively) and lifestyle habits (e.g. physical activity), as well as the tolerability of the capsules.

### Determination of fasting serum lipids and red blood cell (RBC) membrane fatty acid (FA) composition

Fasting venous blood samples were collected in BD Vacationer® Blood Collection Tubes (Becton Dickinson, Heidelberg, Germany). The plasma lipid levels were determined by an external contract laboratory (LADR, Hannover; Germany) at baseline (t_0_), after one week (t_1_) and after twelve weeks (t_12_) of supplementation. RBC membrane FA composition including the omega-3 index, given as EPA and DHA, was analyzed at t_0_ and t_12_ according to the omega-3 index methodology [19]. Results are presented as a percentage of the total FAs identified after response factor correction. The coefficient of variation for EPA and DHA was 5%. Quality was assured according to DIN ISO 15189.

### Statistical analysis of blood levels

Statistical analysis of clinical parameters was processed with SPSS software version 17 (SPSS Inc., Chicago, IL, USA). The results are based on per protocol population, defined as subjects completing all visits not infringing the study protocol, and are presented as mean ± SD (Table 1). Differences between t_0_ and t_12_ were tested within the groups by *t*-test for dependent samples. Group differences were examined with a two-factor variance analysis (ANOVA) combined with a post hoc test of contrast (Scheffé). Additionally, differences between groups were analyzed by covariance analysis (ANCOVA) using the corresponding baseline values as covariates to detect possible effects caused by diverse baseline values. Statistical significance was generally accepted at p<0.05.

**Table 1:** **Anthropometric data, serum lipid levels and red blood cell membrane fatty acid composition of the four treatment groups (FO-N, CO-N, FO-D, CO-D) at baseline (t**_**0**_**) and after supplementation with fish oil or corn oil over twelve weeks (t**_**12**_**)**

**Parameters**	**FO-N (n = 10)**		**CO-N (n = 7)**		**CO-D (n = 8)**		**FO-D (n = 9)**	
	**t**_**0**_	**t**_**12**_	**t**_**0**_	**t**_**12**_	**t**_**0**_	**t**_**12**_	**t**_**0**_	**t**_**12**_
Age [years]	37.50 ± 8.11		37.40 ± 8.30		41.80 ± 8.94		40.20 ± 8.64	
Body height [cm]	180.30 ± 6.29		181.60 ± 7.54		182.80 ± 7.02		180.40 ± 7.55	
Body weight [kg]	78.90 ± 15.25	78.51 ± 16.13	85.80 ± 10.90	84.79 ± 11.19	85.50 ± 14.80	84.53 ± 14.51	94.80 ± 13.20	94.06 ± 13.80
Body mass index [kg/m^2^]	24.20 ± 4.15 ^*c*^	24.13 ± 4.50	25.90 ± 2.83	25.67 ± 2.78	25.40 ± 2.91	25.14 ± 2.86	29.00 ± 2.98 ^*c*^	28.82 ± 3.25
TC [mg/dl]	184.10 ± 13.30 ^*c*^	195.60 ± 26.10	194.00 ± 22.20 ^*d*^	188.90 ± 21.50	261.00 ± 48.10 ^*d*^	257.50 ± 60.30	262.20 ± 61.90 ^*c*^	264.40 ± 49.20
TG [mg/dl]	82.40 ± 35.30 ^*c*^	66.20 ± 16.70 ^*A*^	155.60 ± 53.80	87.40 ± 14.80 ^*A*^	175.00 ± 56.20	186.90 ± 49.60	322.40 ± 258.9 ^*c*^	230.80 ± 147.0 ^*§_T*^
HDL [mg/dl]	59.00 ± 10.40 ^c^	65.20 ± 14.40 ^*§_T*^	52.70 ± 12.70	52.60 ± 11.60	47.90 ± 10.02	47.40 ± 8.21 ^*B*^	45.20 ± 6.50 ^*c*^	50.80 ± 9.43 ^*§B*^
LDL [mg/dl]	108.70 ± 12.80 ^*c_T*^	117.20 ± 22.10	118.10 ± 25.10 ^*d*^	118.60 ± 28.20	178.10 ± 45.40 ^*d*^	172.60 ± 54.40 ^*B_T*^	146.10 ± 5.31 ^*c_T*^	167.40 ± 23.60 ^*§_T B_T*^
LDL/HDL quotient	1.89 ± 0.40 ^*c*^	1.88 ± 0.54	2.41 ± 0.89 ^d^	2.41 ± 0.98	3.74 ± 0.68 ^*d*^	3.60 ± 0.73	3.20 ± 0.58 ^*c*^	3.24 ± 0.73
LA (18:2n-6) [%]*	0.25 ± 0.03	0.19 ± 0.03 ^*§*^	0.23 ± 0.03	0.20 ± 0.05	0.22 ± 0.03	0.18 ± 0.05	0.23 ± 0.03	0.17 ± 0.03 ^*§*^
AA (20:4n-6) [%]*	15.95 ± 0.81 ^*c*^	13.00 ± 0.65 ^*§a*^	15.74 ± 1.48	15.99 ± 1.74 ^*a*^	14.94 ± 1.47	14.88 ± 1.22 ^*b*^	12.82 ± 2.19 ^*c*^	11.98 ± 1.61 ^*b*^
EPA (22:5n-3) [%]*	2.31 ± 0.41	3.74 ± 0.51 ^*§a*^	2.31 ± 0.22	2.44 ± 0.18 ^*a*^	2.09 ± 0.41	2.39 ± 0.35 ^*§b*^	2.00 ± 0.43	3.31 ± 0.26 ^*§b*^
DHA (22:6n-3) [%]*	4.39 ± 0.68	6.87 ± 0.78 ^*§a*^	4.16 ± 1.14	4.09 ± 0.94 ^*a*^	4.28 ± 1.27	4.57 ± 1.10 ^*b*^	3.90 ± 1.42	6.59 ± 0.75 ^*§b*^
Omega-3-index [%]*	5.24 ± 0.70	10.70 ± 1.06 ^*§aA*^	5.08 ± 1.41	4.78 ± 1.16 ^*aA*^	5.27 ± 1.69	5.50 ± 1.57 ^*bB*^	4.90 ± 1.80	9.84 ± 1.26 ^*§bB*^

* percentage of total fatty acids.

*§ *P<0.05 (Changes between baseline and month three were evaluated within groups by Student’s *t *test for dependent samples).

*a *P<0.05 (Changes of means were evaluated between FO-N and CO-N by Student’s *t *test).

*b *P<0.05 (Changes of means were evaluated between FO-D and CO-D by Student’s *t *test).

*c *P<0.05 (Changes of means were evaluated between FO-N and FO-D by Student’s *t *test).

*d *P<0.05 (Changes of means were evaluated between CO-N and CO-D by Student’s *t *test).

*A *P<0.05 (Changes of means were evaluated between FO-N and CO-N group by ANCOVA using baseline as covariate).

*B *P<0.05 (Changes of means were evaluated between FO-D and CO-D group by ANCOVA using baseline as covariate).

_*T *trend of significance (P<0.1).

### Microarray analyses

#### Sample collection

Fasting venous blood samples were collected in PAXgene Blood RNA Tubes (PreAnalytiX, Hombrechtikon, Switzerland) at baseline (t_0_), after one week (t_1_), and after twelve weeks (t_12_) of supplementation to analyze medium- and long-term effects of the FO and CO supplementation on gene expression regulation. For short-term effects, venous blood samples were collected four hours (t_4h_) after the first intake of the capsules. The time point of four hours was selected in accordance with results achieved in a previous study where the strongest postprandial gene expression changes in peripheral blood mononuclear cells of healthy men were observed after four and six hours [20]. The whole blood samples collected were incubated for 24 hours in the PAXgene Blood RNA Tubes at room temperature.

#### Total RNA isolation from human whole blood and RNA purification

The total RNA was isolated with the PAXgene Blood RNA Kit (PreAnalytiX, Hombrechtikon, Switzerland), according to the manufacturer’s recommended procedures. RNA yield was quantified by Nanodrop ND-1000 spectrophotometer (peQLab Biotechnologie GmbH, Erlangen, Germany) measurement. Total RNA was purified with the Globin Clear Kit (Ambon, Applied Bios stems, Darmstadt, Germany), according to the manufacturer’s instructions. The reduction of highly abundant globin mRNA transcripts in whole blood samples is necessary to enable the detection of low-abundance transcripts [21]. The purified RNA was quantified again, and the quality was measured with an Agilent 2100 Bioanalyzer using RNA 6000 Nano Chips and a RNA 6000 Nano Kit (Agilent Technologies, Böblingen, Germany).

#### Sample pooling

Equal amounts of purified RNA samples from each member of the respective group were pooled together. This was done for all different time points (t_0_, t_4h_, t_1_, and t_12_). Therefore, four pool-samples were generated by this process for each group, which results in a total of sixteen pool-samples for the microarray experiments. This approach was chosen to reduce biological inter-individual variability, which is frequent due to variations in relative proportions of specific blood cell subsets, gender, age, and disease state [22].

#### cDNA synthesis and hybridization

First-strand cDNA synthesis and tyramine signal amplification (TSA) were performed using Micromax^TM^ TSA^TM^ Labeling and Detection Kit (Perkin Elmer Life Sciences, Rodgau, Germany) with several protocol modifications. A total amount of 6 μg from every RNA pool, as well as random hexamers primer (Ferment as, St. Leon-Rot, Germany) and oligo dT primer (Roth, Karlsruhe, Germany), were used for the cDNA synthesis, which was facilitated by using Superscript III reverse transcriptase (Invitrogen, Karlsruhe, Germany). The incubation time of two hours was split into two one hour incubations and additional Superscript III was added after first hour. Each RNA pool was synthesized into two different labeled cDNAs, fluorescein-labeled and biotin-labeled cDNA.

After labeling, the cDNA samples were purified with the QIAquick PCR Purification Kit (Qiagen, Hilden, Germany), according to the manufacturer’s instructions. Furthermore, the cDNA samples were first vacuum-dried and then resolved in hybridization buffer (4 x SSPE; 2.5 x Denhardt’s reagent; 30% form amid). After a final degradation step (3 minutes, 95°C), one-tenth of top-block (Sigma-Aldrich, Steinheim, Germany) was added. Equal amounts of biotin-labeled cDNA and fluorescein-labeled cDNA were hybridized simultaneously in one experiment to human whole genome One Array™ Microarrays (Phalanx Biotech Group; Belmont, CA, USA). Hybridizations were carried out overnight at 42°C in hybridization chambers (Eppendorf AG, Hamburg, Germany).

**Figure 2:**
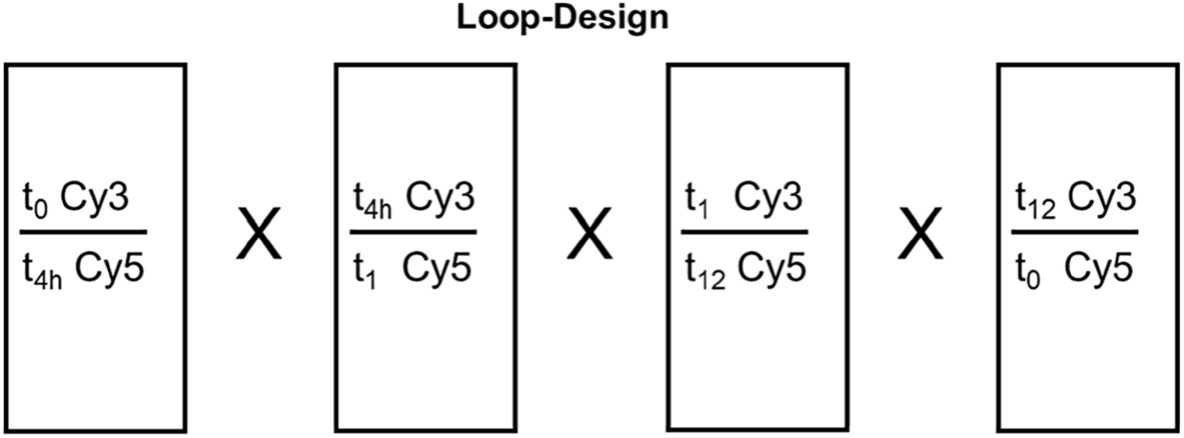
Loop-Design of microarray experiments.

After hybridization, unbound and non-specifically fixed cDNA was removed by stringent washing from the array. Specifically bound fluorescein- and biotin-labeled cDNA were sequentially detected with a series of conjugate reporter molecules according to the TSA process, ultimately with tyramide-Cy3 and tyramide-Cy5. Microarray experiments were performed for each study group in a loop design (Figure 2) to save microarrays and prevent dye-dependent variety effects [23]. 

#### Microarray data analysis

Microarrays were scanned several times with a 4000 B scanner (Axon Instruments, Inc., Union City, CA, USA) and images were quantified using GenePixPro 6.0 Software. The average pixel intensity within each spot was determined and a local background was computed for each spot. Net signal was determined by subtracting local background from the average intensity. Signals not consistently detectable (background corrected signal lower than two times background standard deviation) were excluded from further analysis. Following the primary analysis, data from different scans had to be summarized. The scans first had to be normalized by the sum of all corresponding spot-intensities due to different laser power and photomultiplier-tube settings. Afterwards, data from different scans for each individual spot could be averaged by the mean. The mean of the data for differently labeled targets for each gene on two microarrays was taken. It was assumed that the distribution of the pre-processed data was normal and hence, a standard two-state pooled-variance *t*-test (1% and 5% probability of error) was applied in order to detect differentially expressed genes. Array data were submitted to Gene Expression Omnibus (GEO) which supports minimum information about a microarray experiment (MIAME) [24]. The accession number of the submitted dataset is GSE34898. Genes that were detected as differentially expressed between baseline and measurement time point (t_4h_, t_1_ or t_12_) were subjected to pathway analysis by the Kyoto Encyclopedia of Genes and Genomes (KEGG) database [25].

## Results

### Subject characteristics

At baseline, no significant differences of the mean age, mean weight and the content of EPA and DHA in RBC membranes, as well as in the omega-3 index, were observed between all groups. However, comparison of both FO groups showed that the dyslipidemic FO group had a 4.8 kg/m^2^ higher BMI, higher TC and TG level, lower HDL level, a higher LDL/HDL ratio, and a lower arachidonic acid (AA, 20:4n-6) content in RBC membranes than the normolipidemic FO group (Table 1). Comparisons of both CO groups showed higher LDL and TC levels, as well as a higher LDL/HDL ratio, in the dyslipidemic CO group than in the normolipidemic CO group.

All 40 subjects (20 normolipidemic and 20 dyslipidemic men) completed the study. However, it was necessary to exclude the RNA samples of ten subjects from the microarray experiments and data analysis due to several reasons: Low RNA yield (four subjects), consumption of alcohol with effects on serum TG levels (two subjects), consumption of medication that lead to exclusion (three subjects), and highly elevated TG level (> 1000 in one subject). Therefore, RNA pools were generated and data were analyzed from nine (FO-N), six (CO-N), eight (CO-D), and seven (FO-D) subjects for each investigation time point (Figure 1).

### Changes of blood lipids, FA composition of RBC membranes and omega-3 index

The effects of the supplementation with either FO or CO in normo- and dyslipidemic subjects on serum lipids and FA composition of RBC membranes are shown in Table 1. Blood lipids showed only slight differences between t_0_ and t_12_. A significant increase in HDL levels was observed in the FO-D group, whereas a tendency for increased HDL levels was seen in the FO-N group. Furthermore, trends for an increased LDL and decreased TG level were observed in the FO-D group. Comparison of the t_12_ values between the FO and CO groups with a covariance analysis (ANCOVA) using t_0_ values as covariates, showed lower TG levels in the FO-N group than in the CO-N group and higher HDL levels, as well as a trend of lower LDL levels, in the FO-D group than in the CO-D group.

Several significant differences could be observed in the FA composition of RBC membranes and the omega-3 index between the groups. Within both FO groups, the percentage of EPA and DHA, as well as the omega-3 index in RBC membranes, significantly increased after twelve weeks of supplementation (p<0.001). Additionally, the FO-N group showed a significant decrease of the percentage of AA and LA in RBC membranes. Within both CO groups, no statistical differences between t_0_ and t_12_ values could be detected, except the increase of the percentage of EPA in RBC membranes in the CO-D group. Group comparisons of the t_12_ values between the FO and CO groups showed significantly higher percentages of EPA, DHA and omega-3 index, as well as lower percentages of AA in RBC membranes in both the FO-N and FO-D group in comparison to the respective CO groups.

### Microarray analyses

#### Number of regulated genes

**Figure 3:**
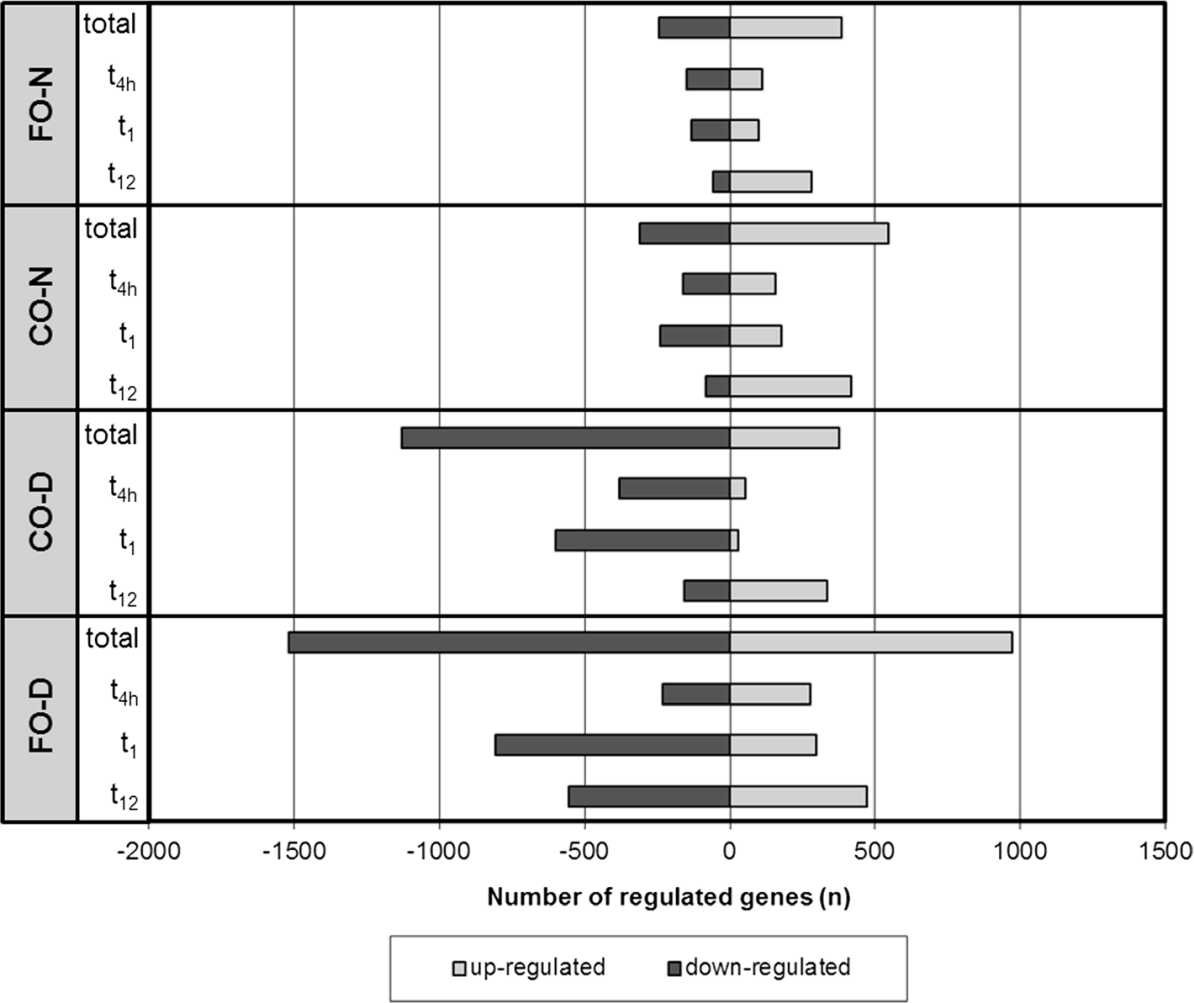
**Number of regulated genes. **Number of genes that were regulated in the normolipidemic fish oil group (FO-N), normolipidemic corn oil group (CO-N), dyslipidemic corn oil group (CO-D), and dyslipidemic fish oil group (FO-D) after four hours (t_4h_), one week (t_1_) and twelve weeks (t_12_) of supplementation. Total regulated genes summarize all genes regulated during the different time points, without doubles, respectively.

The total number of regulated genes of each study group was determined and compared between groups. Both dyslipidemic study groups showed a higher number of regulated genes than normolipidemic study groups, independent of the type of supplemented oil (Figure 3). The differences in the total number of regulated genes between the two CO groups are small, whereas both FO groups showed substantial differences. A further determination of the direction of regulated genes in total within each group discovered a higher number of down-regulated genes in both dyslipidemic groups (CO-D and FO-D). In contrast, a higher number of up-regulated genes were observed in both normolipidemic groups (CO-N and FO-N). Another observation was made concerning the pattern of up- and down-regulated genes within each group depending on the time point of investigation. Whereas the pattern of up- and down-regulated genes was similar at the first two early time points (t_4h_ and t_1_), the pattern changed into the opposite direction after twelve weeks of supplementation (t_12_). This observation was made in all groups, except the FO-D group.

#### Pathway analyses

Pathway analysis of the regulated genes were performed for each group and time point. All pathways with regulated genes were attributed to processes according to the KEGG database (metabolism; genetic information processing, environmental information processing, cellular processes, organismal systems and human diseases) [25] to analyze the influence of the supplemented oil type on gene expression changes in different processes. Furthermore, the number of regulated genes in pathways belonging to different processes was determined for each group and compared as a percentage of total regulated genes between groups. Thus, the influence of the supplemented oil type on different processes could be investigated. Table 2 shows processes where a higher number of genes in both FO groups were regulated at the different time points compared to the corresponding CO groups. It was noticeable that the lipid metabolism was regulated at all time points after FO supplementation. A greater number of processes were regulated in both FO groups after twelve weeks (t_12_), including, for example, lipid metabolism, the immune system and cardiovascular diseases. 

**Table 2:** **Selected processes dominantly regulated in dys- and normolipidemic subjects in response to fish oil supplementation for four hours (t**_**4h **_**), one week (t**_**1**_**) and twelve weeks (t**_**12**_**) compared to corn oil supplementation**

**t**_**4h**_	**t**_**1**_	**t**_**12**_
Cell Communication	Carbohydrate Metabolism	Amino Acid Metabolism
Cell Growth and Death	Cardiovascular Diseases	Biosynthesis of Other
Circulatory System	Circulatory System	Secondary Metabolites
Digestive System	Digestive System	Cardiovascular Diseases
Endocrine System	Energy Metabolism	Cell Growth and Death
Excretory System	Folding, Sorting and	Endocrine System
Folding, Sorting and	Degradation	Energy Metabolism
Degradation	Lipid Metabolism	Excretory System
Immune System	Signal Transduction	Immune System
Lipid Metabolism	Signalling Molecules and	Immune System Diseases
Nervous System	Interaction	Lipid Metabolism
Signalling Molecules and Interaction	Xenobiotics Biodegradation and Metabolism	Metabolic Diseases
		Metabolism of Cofactors and Vitamins
		Metabolism of Other Amino Acids
		Nervous System
		Nucleotide Metabolism
		Sensory System

The selection was performed as follows: The number of regulated genes within pathways was counted for each group. Pathways were assigned to corresponding processes and the number of regulated genes in these pathways was summed up. The resulting data represent the number of regulated genes in different processes for each group. These numbers were compared between groups and those processes were selected where both fish oil treatment groups (FO-D, FO-N) showed more regulated genes compared with the corn oil treatment groups (CO-D, CO-N).

Further analyses concentrated on the long-term effects after twelve weeks of FO supplementation (t_12_). Therefore, corresponding pathways and regulated genes from three exemplary processes that were dominantly regulated in both FO groups after twelve weeks (lipid metabolism, the immune system, cardiovascular diseases) were selected and listed for the FO-D group in Table 3. In some pathways of the lipid metabolism, genes were mainly down-regulated (FA synthesis,-metabolism and elongation, AA-, LA- and alpha linolenic acid metabolism), whereas in others pathways, genes were mainly up-regulated (glycerolipid and glycerophospholipid metabolism). The immune system-related pathway of complement and coagulation cascade, as well as the two pathways related to cardiovascular diseases, showed mainly down-regulated genes.

**Table 3:** Selected pathways and genes dominantly regulated in dyslipidemic subjects (FO-D) in response to fish oil supplementation for twelve weeks

	**Pathway**	**Gene name**	**Gene symbol**	**Entrez_ID**	**Ratio t**_**12**_**: t**_**0**_
**Lipid metabolism**	Fatty acid biosynthesis	acetyl-CoA carboxylase beta	ACACB	32	-4.67
	Fatty acid elongation in mitochondria	hydroxyacyl-CoA dehydrogenase/3-ketoacyl-CoA thiolase/enoyl-CoA hydratase (trifunctional protein), alpha subunit	HADHA	3030	-3.70
		hydroxyacyl-CoA dehydrogenase	HADH	3033	-2.34
	Fatty acid metabolism	enoyl-CoA delta isomerase 2	ECI2	10455	-4.54
		enoyl-CoA delta isomerase 1	ECI1	1632	6.02
		hydroxyacyl-CoA dehydrogenase/3-ketoacyl-CoA thiolase/enoyl-CoA hydratase (trifunctional protein), alpha subunit	HADHA	3030	-3.70
		hydroxyacyl-CoA dehydrogenase	HADH	3033	-2.34
		acyl-CoA dehydrogenase, short/branched chain	ACADSB	36	-13.69
		aldehyde dehydrogenase 2 family (mitochondrial)	ALDH2	217	3.26
	Glycerolipid metabolism	1-acylglycerol-3-phosphate O-acyltransferase 6	AGPAT6	137964	2.67
		diacylglycerol kinase, alpha 80 kDa	DGKA	1606	2.47
		diacylglycerol kinase, eta	DGKH	160851	-3.05
		diacylglycerol kinase, theta 110 kDa	DGKQ	1609	3.40
		aldehyde dehydrogenase 2 family (mitochondrial)	ALDH2	217	3.26
		monoacylglycerol O-acyltransferase 3	MOGAT3	346606	-3.48
	Glycerophospholipid metabolism	1-acylglycerol-3-phosphate O-acyltransferase 6	AGPAT6	137964	2.67
		phospholipase B1	PLB1	151056	11.68
		diacylglycerol kinase, alpha 80 kDa	DGKA	1606	2.47
		diacylglycerol kinase, eta	DGKH	160851	-3.05
		diacylglycerol kinase, theta 110 kDa	DGKQ	1609	3.40
		phospholipase A2, group IIE	PLA2G2E	30814	2.51
		phosphatidylserine synthase 1	PTDSS1	9791	-2.84
	Arachidonic acid metabolism	phospholipase B1	PLB1	151056	11.68
		gamma-glutamyltransferase 5	GGT5	2687	7.98
		glutathione peroxidase 3 (plasma)	GPX3	2878	-2.15
		glutathione peroxidase 5	GPX5	2880	-2.41
		phospholipase A2, group IIE	PLA2G2E	30814	2.51
	Linoleic acid metabolism	phospholipase B1	PLB1	151056	11.68
		phospholipase A2, group IIE	PLA2G2E	30814	2.51
	Alpha-linolenic acid metabolism	phospholipase B1	PLB1	151056	11.68
		phospholipase A2, group IIE	PLA2G2E	30814	2.51
**Immune system**	Complement and coagulation cascades	complement component (3b/4b) receptor 1	CR1	1378	-3.37
		coagulation factor III (thromboplastin, tissue factor)	F3	2152	-4.23
		fibrinogen gamma chain	FGG	2266	-12.30
		serpin peptidase inhibitor, clade C (antithrombin), member 1	SERPINC1	462	3.23
**Cardiovascular diseases**	Arrhythmogenic right ventricular cardiomyopathy (ARVC)	desmocollin 2	DSC2	1824	-2.50
		calcium channel, voltage-dependent, gamma subunit 5	CACNG5	27091	-9.92
		integrin, alpha 3	ITGA3	3675	-4.32
		transcription factor 7-like 1	TCF7L1	83439	-4.93
	Dilated cardiomyopathy	adenylate cyclase 7	ADCY7	113	-2.13
		calcium channel, voltage-dependent, gamma subunit 5	CACNG5	27091	-9.92
		integrin, alpha 3	ITGA3	3675	-4.32
		myosin, light chain 2, regulatory, cardiac, slow	MYL2	4633	2.68

Generally, the majority of regulated genes in all groups were detected in pathways related to the immune system. It is known that FO supplementation provokes immune-modulatory and anti-inflammatory effects. Therefore, regulated genes related to inflammation and immune response, were individually selected for the FO-D group to show the direction of regulation (Table 4). This examination showed that a greater number of pro-inflammatory genes were down-regulated after twelve weeks of supplementation, while a higher number of immune response related genes were up-regulated. However, roughly the same number of anti-inflammatory genes was up- and down-regulated.

**Table 4:** Selection of inflammation and immune response related genes regulated in dyslipidemic subjects after supplementation with fish oil for twelve weeks

**Gene name**	**Gene symbol**	**Entrez_ID**	**Ratio t**_**12**_**: t**_**0**_
**Pro-inflammatory genes**			
Nitric oxide synthase, inducible	NOS2	4843	-4.77
C-C chemokine receptor type 3	CCR3	1232	-4.49
Tumour necrosis factor receptor superfamily member 18 Precursor	TNFRSF18	8784	-2.07
Interleukin-31 receptor A Precursor	IL31RA	133396	-4.74
72 kDa type IV collagenase Precursor	MMP2	4313	-6.16
Interleukin-8 Precursor	IL8	3576	-5.67
Tumour necrosis factor receptor superfamily member 5 Precursor	CD40	958	-4.72
Interleukin-2 receptor subunit beta Precursor	IL2RB	3560	-3.22
Interleukin-3 receptor subunit alpha Precursor	IL3RA	3563	-2.22
Stromelysin-1 Precursor	MMP3	4314	-2.17
C-C motif chemokine 5 Precursor	CCL5	6352	2.24
CD97 antigen Precursor	CD97	976	2.26
Prostaglandin D2 receptor	PTGDR	5729	4.47
Integrin alpha-L Precursor	ITGAL	3683	5.07
Arachidonate 5-lipoxygenase-activating protein (FLAP)	ALOX5AP	241	6.97
**Anti-inflammatory genes**			
C-X-C motif chemokine 11 Precursor	CXCL11	6373	-2.98
NF-kappa-B-repressing factor	NKRF	55922	-82.11
NF-kappa-B inhibitor-like protein 2	NFKBIL2	4796	-3.30
Cytokine receptor-like factor 1 Precursor	CRLF1	9244	-3.07
Lipopolysaccharide-induced tumour necrosis factor-alpha factor	LITAF	9516	3.06
Interferon regulatory factor 1	IRF1	3659	3.16
Interferon-alpha/beta receptor alpha chain Precursor	IFNAR1	3454	5.13
Suppressor of cytokine signalling 6	SOCS6	9306	6.01
**Immune response related genes**			
Suppressor of cytokine signalling 2	SOCS2	8835	-6.05
Disintegrin and metalloproteinase domain-containing protein 10 Precursor	ADAM10	102	-5.23
A disintegrin and metalloproteinase with thrombospondin motifs 10 Precursor	ADAMTS10	81794	-3.14
Nitric oxide synthase, endothelial	NOS3	4846	-2.55
SL cytokine Precursor	FLT3LG	2323	-2.46
Prostaglandin E2 receptor EP3 subtype	PTGER3	5733	-2.21
CD99 antigen-like protein 2 Precursor	CD99L2	83692	2.18
Cytokine receptor common gamma chain Precursor	IL2RG	3561	2.85
Monocyte differentiation antigen CD14 Precursor	CD14	929	2.88
Chemokine-like factor (C32)	CKLF	51192	11.73
T-cell surface glycoprotein CD3 delta chain Precursor	CD3D	915	7.65
Tumour necrosis factor receptor superfamily member 1B Precursor	TNFRSF1B	7133	2.51
B-lymphocyte antigen CD20	MS4A1	931	2.12
Interleukin-31 receptor A Precursor	IL31RA	133396	5.72
Intercellular adhesion molecule 3 Precursor	ICAM3	3385	5.85
CD320 antigen Precursor	CD320	51293	8.17
CD2 antigen cytoplasmic tail-binding protein 2	CD2BP2	10421	8.77
T-cell antigen CD7 Precursor	CD7	924	2.30
C-C chemokine receptor-like 2 (Putative MCP-1 chemokine receptor)	CCRL2	9034	17.55

## Discussion

It is known from numerous *in vitro* and *in vivo* studies that FO and its general n-3 PUFAs, EPA and DHA, regulate the expression of genes, but human intervention studies investigating the effect of FO on whole genome gene expression are lacking. Our study aimed to investigate two main objectives: The first intention was to investigate the effects of FO supplementation on gene expression changes in dyslipidemic subjects in comparison to normolipidemic subjects. The second objective was to compare the effects of FO with CO, which is used as placebo in many intervention studies, investigating the physiological effects of FO. To the best of our knowledge, this is the first randomized intervention study disclosing gene expression profiles on the whole genome level under these specific conditions. In general, we anticipated regulative effects of FO on the expression of genes associated with the immune system and the lipid metabolism, especially in dyslipidemic subjects.

### Serum lipid and omega-3 index levels

As expected, the FO supplementation of dyslipidemic subjects was followed by an increase in HDL levels and a decrease in TG levels after twelve weeks, although not significant due to the low group size. There was little or no effect on the lipid levels in normolipidemic subjects after FO or CO supplementation. The omega-3 index increased significantly in both FO treatment groups from levels ~ 5% to levels of ~10% after twelve weeks of supplementation, which is a shift from an unfavorable area in view of the cardiovascular risk to optimal levels of 8% or greater [26]. Moreover, the omega-3 index increased in all subjects of the two FO groups, indicating an efficient uptake and subsequent incorporation of EPA and DHA in tissue membranes. It can, therefore, be assumed that changes observed in gene expression in the FO supplementation groups are the result of the treatment.

### Normolipidemic vs. dyslipidemic subjects

Gene expression profiles of normo- and dyslipidemic subjects differed substantially after FO supplementation. In the dyslipidemic study group, considerably more genes were regulated after FO supplementation, whereas genes were mainly down-regulated. Although the reason for this finding cannot be clarified here, it might be partly explained by the interrelation between inflammation and dyslipidemia [18]. Several studies have demonstrated that a dyslipidemic – in particular hypertriglyceridemic – state is accompanied by an induction of pro-inflammatory pathways [27,28], while n-3 PUFAs are known to suppress these pathways [9]. Indeed, a number of pro-inflammatory genes were down-regulated in dyslipidemic subjects after FO supplementation, including members of the TNFα pathway. However, besides the regulated genes involved in inflammatory pathways, it is apparent that a large number of other genes were regulated in dyslipidemic subjects in response to FO treatment, suggesting a strong regulative effect of FO in people with dyslipidemia. It needs to be clarified in future studies if some of these genes are associated with the known beneficial effects FO or n-3 PUFAs in dyslipidemia.

In contrast to dyslipidemic subjects, a higher number of genes were up-regulated in normolipidemic subjects in response to FO supplementation. This finding corresponds with the outcomes of two intervention studies investigating the effect of FO supplementation on whole genome expression in healthy subjects [16,17]. The study of Gorjão and co-workers was conducted with ten men aged between 25 and 45 years supplemented with 1.62 g DHA and 0.78 g EPA per day over a period of eight weeks. The design of this study is comparable with our study, except for the sample material used for RNA isolation (fractionated lymphocytes vs. whole blood). Different RNA sources could yield different expression patterns, and cell fractioning itself could alter the gene expression pattern [29]. Nevertheless, both studies provided similar results. Gorjão and co-workers discovered 71 up- and 6 down-regulated genes by macroarray analysis. Our normolipidemic FO group showed 627 total regulated genes, 383 of which were up-regulated and 244 down-regulated. Therefore, both studies indicate that genes in normolipidemic subjects are mainly up-regulated after FO supplementation. In the study by Bouwens and colleagues, a total of 111 men and woman aged between 66 and 80 years were supplemented with 1.09 g EPA and 0.85 g DHA per day over 26 weeks. There were great differences in the study design compared to our study with regard to gender (men and woman vs. men), subject age (older vs. middle aged), daily n-3 PUFAs intake (1.94 g vs. 2.7 g/d EPA + DHA), and sample material used for RNA isolation (peripheral blood mononuclear cells vs. whole blood), which makes it difficult to compare the results. However, the number of total regulated genes in both studies was in the same order of magnitude. Bouwens and co-workers identified a total of 1040 regulated genes, with 537 up-regulated and 503 down-regulated genes.

### Fish oil vs. Corn oil

CO serves as placebo in many FO supplementation studies based on the assumption that CO is inert without biological effects. However, CO is rich in the n-6 PUFA LA, a precursor for AA. Thus, CO similarly contains FAs, capable of directly regulating gene expression or serving as precursors for other bioactive lipid mediators which regulate gene expression. It was, therefore, our intention to enlighten the possible regulative effects of CO on gene expression. The administration of six CO capsules provided three grams of LA per day, which is less than a quarter of the usual dietary intake of LA, and considerably less than a high consumption of vegetable oil. In view of this small amount and the low conversion rate of LA to AA, we assumed that the effect of LA on gene expression would be infinitesimal. Surprisingly, our data revealed that the administration of even small amounts of LA caused changes in gene expression patterns. However, during the evaluation of the study data, we focused on the specific effects of FO on gene expression.

Our data demonstrated substantial differences in gene expression regulation between FO and CO supplementation. Pathway analysis showed that considerably more pathways were dominantly regulated in the FO groups compared to the CO groups (in both normolipidemic and dyslipidemic subjects). Additionally, significantly more genes in these pathways and metabolisms were shown to be regulated in dyslipidemic subjects after FO supplementation compared to normolipidemic subjects, suggesting a more pronounced regulative potential of FO in dyslipidemic subjects. Metabolisms that were dominantly affected by FO include, for example, the immune system, lipid metabolism and CVD.

#### Immune system metabolism

Both FO and CO administration resulted in the regulation of genes associated with the immune system and infectious diseases, which is expected in view of the RNA source used. RNA was isolated from whole blood, including leukocytes, which are mainly involved in the immune system and pathogen defense. Therefore, it is not surprising that a great number of regulated genes are involved in immune system related pathways. However, comparing the effect of FO and CO supplementation on the expression of immune system related genes, it appears that FO regulates significantly more genes in specific pathways compared to CO, indicating the immune-modulatory capability of FO and its bioactive FAs, EPA and DHA. Generally, EPA and DHA have anti-inflammatory properties by increasing the production of potent bioactive lipid mediators (protecting and resolving), and inhibiting the formation of n-6 FA-derived pro-inflammatory eicosanoids (e.g. prostaglandine E2 and Leukotriene B4) [30]. Together, these effects directly or indirectly suppress the activity of nuclear transcription factors, such as nuclear factor kappa b, which controls the expression of a variety of pro-inflammatory and pro-atherogenic genes, including those encoding for interleukin (IL)-1, IL-6, IL-8, tumor necrosis factor alpha (TNFα), E-selecting, vascular cell adhesion molecule-1, and cyclooxygenase-2 [11,30].

With a few exceptions, our data revealed that none of the key regulators mentioned above were regulated after FO supplementation, either in dyslipidemic or in normolipidemic subjects (IL-8 was down-regulated after one week and twelve weeks in dyslipidemic FO-supplemented subjects, as well as IL-1 receptor, type II after one week). However, a number of inflammation and immune response related genes were regulated after twelve weeks of FO supplementation, especially in dyslipidemic subjects. The ratios demonstrate that more pro-inflammatory genes were down-regulated than up-regulated, whereas the rate of up- and down-regulation was balanced for anti-inflammatory genes. Moreover, pathway analyses showed that genes of the complement and coagulation cascades (complement component [3b/4b] receptor 1, coagulation factor III [thromboplastin, tissue factor], fibrinogen gamma chain) were mainly down-regulated in dyslipidemic subjects supplemented with FO. Since the blood coagulation cascade is evolutionarily closely related to the innate immune response, its pathways are assigned to the immune system. The coagulation pathway is essential for clot formation and the prevention of excessive bleeding. A dysregulation of the cascade activities can result in clinical manifestations of several diseases with critical thrombotic and/or inflammatory complications [31]. Our results underline the well-known effect of FO to diminish the coagulant activity by the lowering of several coagulation factors and by reducing the capability of plasma to support thrombin generation, especially in patients with hyperlipidemia [32].

#### Lipid metabolism

As expected, pathway analyses revealed that the effect of FO supplementation on the expression of genes involved in lipid metabolism was more pronounced compared to CO. Similarly, the regulatory effect of FO supplementation was markedly stronger among dyslipidemic subjects compared to normolipidemic subjects relating to the number of regulated genes. The liver plays a central role in lipid metabolism and n-3 PUFAs have been shown to regulate hepatic gene expression by targeting several transcriptional regulatory networks [9]: For example, n-3 PUFAs regulate several inflammation molecules, including serum amyloid A, TNFα and IL-6 [33]. These inflammation mediators modulate the expression of many lipid metabolism-related genes, for example, by suppressing the expression of perilipin, sterol regulatory element binding protein-1 and lipoprotein lipase. Together, these regulatory pathways result in induced lipolysis and reduced lipogenesis [33]. Besides the down-regulation of FA synthesis gene expression, n-3 PUFAs up-regulate gene expression involved in FA oxidation, which is triggered by an activation of the transcription factor peroxisome proliferator activated receptors alpha (PPARα) [9].

Again, none of the lipid metabolism-related genes mentioned above were regulated in the present study after 12 weeks of supplementation, probably because the compartment examined (whole blood) is inappropriate to reflect the transcriptional profile of the liver. However, FO supplementation provoked a regulation of several lipid metabolism-associated pathways in dyslipidemic subjects in contrast to normolipidemic subjects, where only a few genes were regulated. This finding emphasizes the regulating effect of n-3 PUFAs on the lipid metabolism in dyslipidemic conditions. Genes, for example, in pathways related to FA metabolism and FA elongation in mitochondria, including genes coding several enzymes, were mainly down-regulated in FO supplemented dyslipidemic subjects. These enzymes (several dehydrogenises and an isomerase) are mainly promoters of mitochondrial FA oxidation. This down-regulation is in contrast to studies, which showed an increased peroxisomal FA oxidation in rats in response to FO administration, while the effect of FO on mitochondrial ß-oxidation was inconsistent [34]. The observed reduced FA oxidation after 12 weeks of FO treatment in this study may be the result of increasing levels of oxidized n-3 PUFAs, which are prone to oxidation. Subsequently, the resulting oxidative stress may lead to an induction of antioxidative mechanisms, which in turn reduce oxidation. The finding that PPARα was up-regulated one week after FO capsule ingestion in dyslipidemic subjects (data not shown) support this hypothesis.

We observed a down-regulation of the gene coding for acetyl-CoA carboxylase beta, which is one of the key enzymes in FA biosynthesis, indicating a reduced lipogenesis. This finding may partly explain the reduced TG levels observed in this study. In the same group, several genes in pathways involved in FA metabolism were regulated. Interestingly, genes coding for Phospholipase A2, group IIE (PLA2G2E), a member of the secreted Phospholipase A2 [sPLA2] family) and Phospholipase B1 (PLB1) were up-regulated. The regulatory functions of PLA2G2E have not been completely uncovered. It is known that PLA2G2E catalyzes the hydrolysis of the 2-acyl groups in 3-sn-phosphoglycerides in membranes and could promote inflammation, since the release of AA is the first step in the AA breakdown pathway, which can be metabolized to several inflammatory and thrombogenic eicosanoids (prostaglandines and Leukotrienes) by the activity of cyclooxygenase and lipoxygenase [35]. The up-regulation of PLA2G2E observed could also point to an intensified exchange of membrane bound AA in favor of EPA and DHA, which is indeed reflected in the decrease in AA levels and concomitant increase in EPA and DHA levels in RBC membranes observed.

In addition, an up-regulation of several genes related to glycerolipid metabolism was observed in FO supplemented dyslipidemic subjects. Glycerolipids are essential components of membranes and an up-regulation of the glycerolipid metabolism indicates remodeling activities of the membrane. Finally, several genes of the glycerophospholipid metabolism were up-regulated in FO supplemented dyslipidemic subjects. Glycerophospholipids, also referred to as phospholipids, are key components of the lipid bilayer of biological membranes and constitute the binding site for EPA and DHA, which are integrated in the membrane. An up-regulation of the glycerophospholipid metabolism results in an increased de novo phospholipid biosynthesis, enabling the incorporation of EPA and DHA into the membrane [36].

#### CVD metabolism

Pathway analysis showed that FO supplementation induced the regulation of pathways involved in specific CVD related metabolisms, especially among dyslipidemic subjects. Several genes from pathways involved in arrhythmogenic right ventricular cardiomyopathy (ARVC) and dilated cardiomyopathy (DCM) were mainly down-regulated. ARVC and DCM belong to cardiomyopathies, a group of diseases that primarily affect the myocardium. While ARVC is characterized by a fibro-fatty replacement of right ventricle myocardium, DCM is a myocardial disease with dilated left ventricle myocardium impairing the systolic pump function of the heart. ARVC and DCM may cause ventricular tachyarrhythmias, blood clots or sudden death. Although the aetiopathogenesis, including the role of dyslipidemia in ARVC and DCM, is largely unknown [37], inflammatory processes are likely to be involved [38,39]. The down-regulating effect of FO on genes involved in ARVC and DCM pathways observed may be the result of diverse regulatory effects on lipid metabolism and anti-inflammatory processes. However, the effect of n-3 PUFA supplementation on the pathogenesis of ARVC and DCM in humans is unknown. An animal study showed that FO supplementation reduces arrhythmia in boxers (*Canis lupus)* with ARVC [40]. In a recent intervention study with non-ischemic DCM patients, it was shown that n-3 PUFA treatment increased left ventricle systolic function and functional capacity [41].

### Strengths and limitations

Strengths: The methodological approach of this study was carefully elaborated. The use of whole blood for RNA isolation is advantageous in view of the easy sample collection and the prevention of altered gene expression patterns which emerge during cell fractionation steps [29]. In addition, the pooling of RNA samples reduces inter-individual variation, enabling one to focus on the characteristics of a population in contrast to an individual level [42]. Moreover, only men were enrolled in the study population. Women are subjected to several hormonal changes which involve individual gene expression changes and hamper the attribution of observed effects to treatment.

Limitations: The study has a number of potential limitations, for example, the small sample size. Moreover, nine subject samples had to be excluded from microarray analysis. To minimize the already high effort for the participants, it was desisted from obtaining multiple baseline samples, which is recommended due to heavy fluctuations in lipid – especially TG – levels. The effect of FO on gene expression was compared to CO, which is often used in n-3 PUFA supplementation studies as a placebo control. It would have been instructive to examine the gene expression of a third untreated study group. However, this additional expenditure would have gone beyond the scope of our study.

## Conclusion

This is the first study showing significant differences in gene expression profiles between normo- and dyslipidemic subjects after FO supplementation. Dyslipidemic subjects presented substantially more regulated genes and pathways, which were mainly down-regulated. Several of the pathways that were especially regulated in dyslipidemic subjects in response to FO supplementation are related to the immune system, inflammation, lipid metabolism, and CVD. In particular, several genes involved in FA metabolism were down-regulated, emphasizing the regulating effect of n-3 PUFA on the lipid metabolism in dyslipidemic conditions. Further studies combining genetic with physiological endpoints need to clarify the mechanisms by which n-3 PUFAs trigger gene regulation and affect various regulatory networks. The disentanglement of such interferences may also explain the beneficial effects of n-3 PUFAs on dyslipidemia, atherosclerosis and CVD in many experimental models and clinical conditions.

## Abbreviations

AA: Arachidonic acid (20:5n-6); ARVC: Arrhythmogenic right ventricular cardiomyopathy; BMI: Body mass index; CO: Corn oil; CO-D: Dyslipidemic corn oil group; CO-N: Normolipidemic corn oil group; CVD: Cardiovascular disease; DCM: Dilated cardiomyopathy; DHA: Docosahexaenoic acid (22:6 n-3); EPA: Eicosapentaenoic acid (20:5 n-3); FA: Fatty acid; FO: Fish oil; FO-D: Dyslipidemic fish oil group; FO-N: Normolipidemic fish oil group; HDL: High density lipoprotein; IL: Interleukin; LA: Linoleic acid (18:2 n-6); LDL: Low density lipoprotein; n-3: Omega-3; n-6, Omega-6; PLA2G2E: Phospholipase 2 group 2 E; PLB1: Phospholipase B1; PUFA: Polyunsaturated fatty acid; RBC: Red blood cell; TC: Total cholesterol; TG: Triacylglycerol; TNFα: Tumor necrosis factor alpha.

## Competing interests

The authors declare that they have no competing interests.

## Authors’ contributions

All authors have read and approved the final manuscript. SS was involved in the study and experimental design, data analysis, interpretation, and manuscript writing. The study was mainly performed by SS. FS was involved in the experimental design and informed advice. KOM was involved in the experimental design, data analysis and manuscript editing. JPS was involved in study design, data interpretation and manuscript writing. The group leader of the Institute of Technical Chemistry, TS, was involved in the study design and manuscript editing. The group leader of the Institute of Food Science and Human Nutrition, AH, was involved in the study design and manuscript editing. Both JPS and AH were coordinators of the study. All authors read and approved the final manuscript.
